# Supplementary figures and images for: Interspecies differences in the transcriptome response of corals to acute heat stress
Source: PeerJ. 2024 Dec 10;12:e18627. doi: 10.7717/peerj.18627 (PMC11639872; doi:10.7717/peerj.18627)

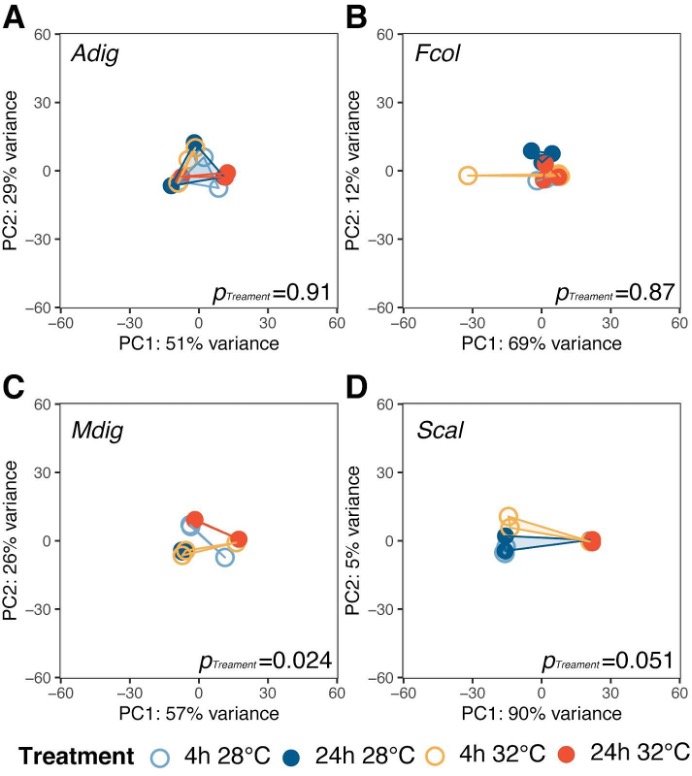

Supplement: Supplemental Information 1 — Principal component analysis (PCA) and gene expression plasticity plots of symbiont-derived transcriptome profiles for (A) A. digitifera, (B) F. colemani, (C) M. digitata, and (D) S. caliendrum in different treatments. The x- and y-axes represent the % variance explained by the first two principal components. [file peerj-12-18627-s001.jpg]

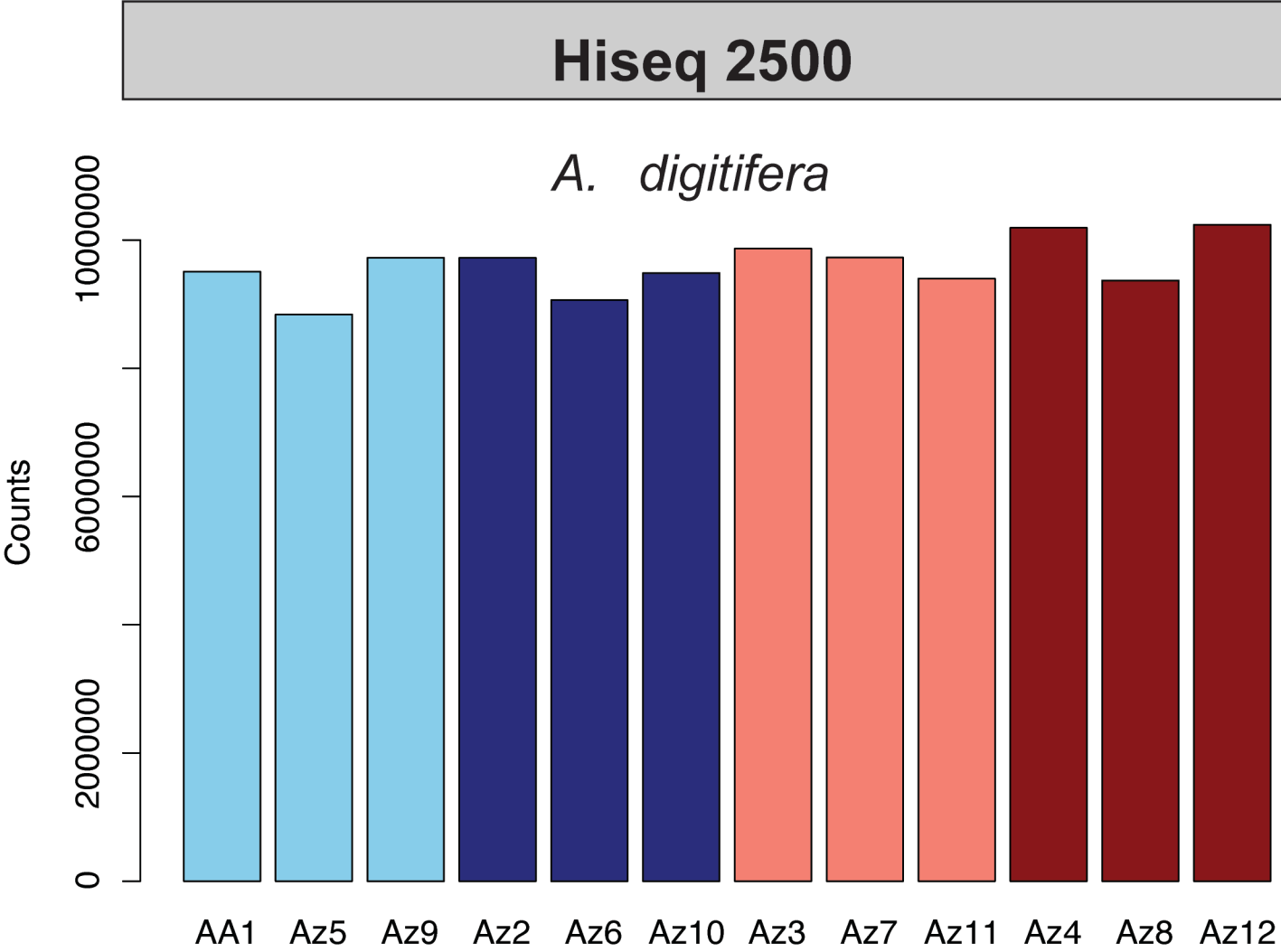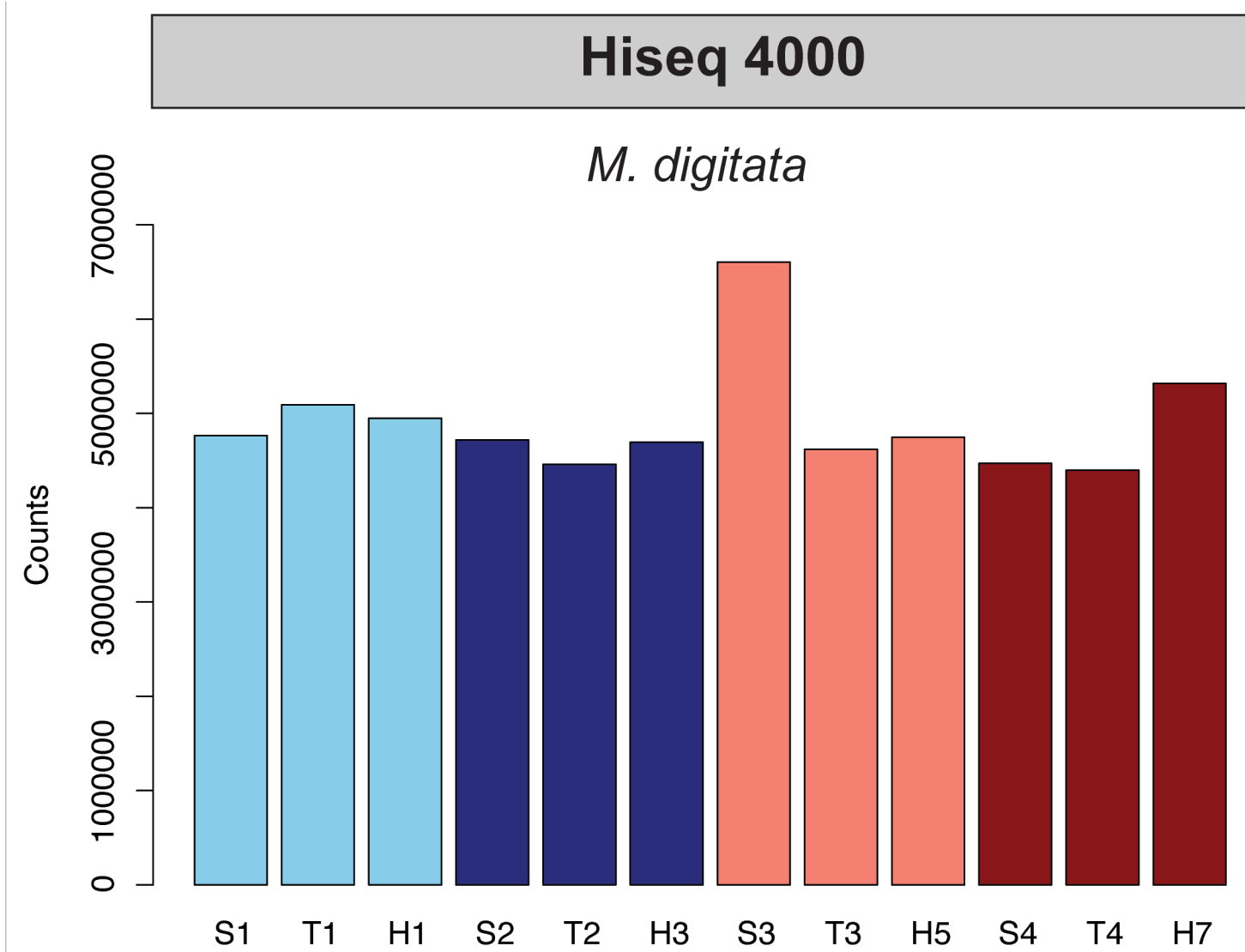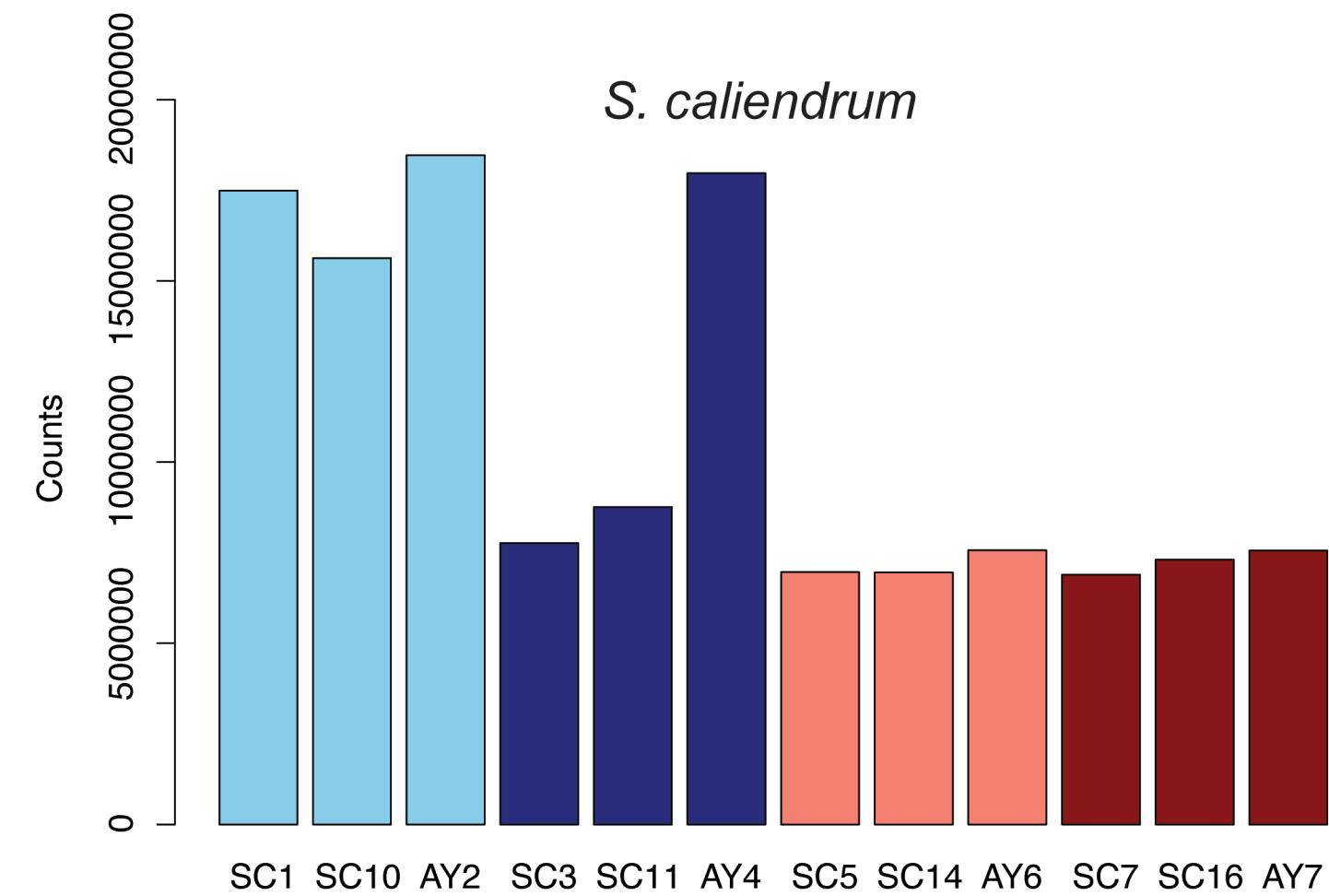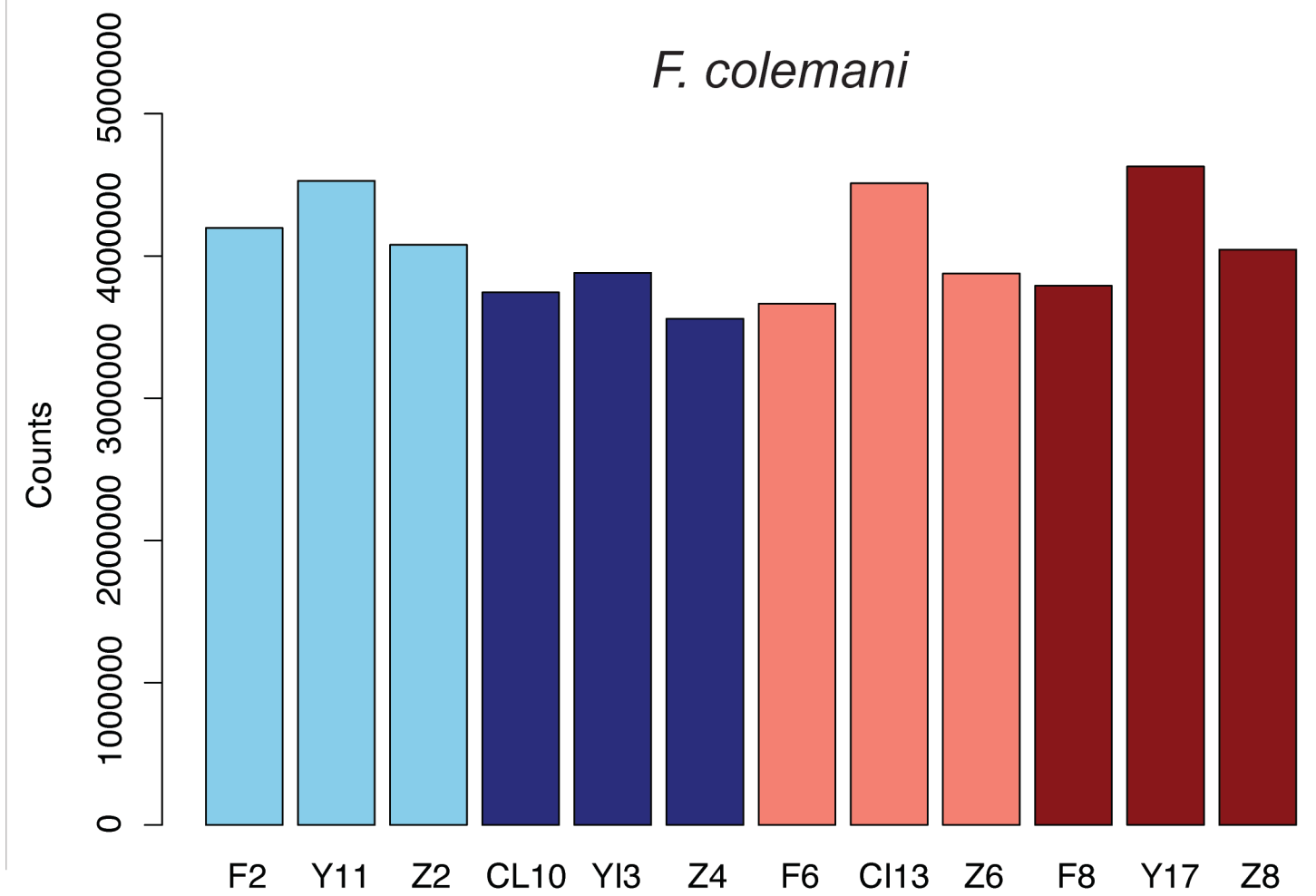

Supplement: Supplemental Information 2 — Number of raw sequence reads per library for each coral species. Acropora digitifera and Seriatopora caliendrum were sequenced on HiSeq2500, while Montipora digitata and Favites colemani were sequenced on HiSeq4000. [file peerj-12-18627-s002.pdf]

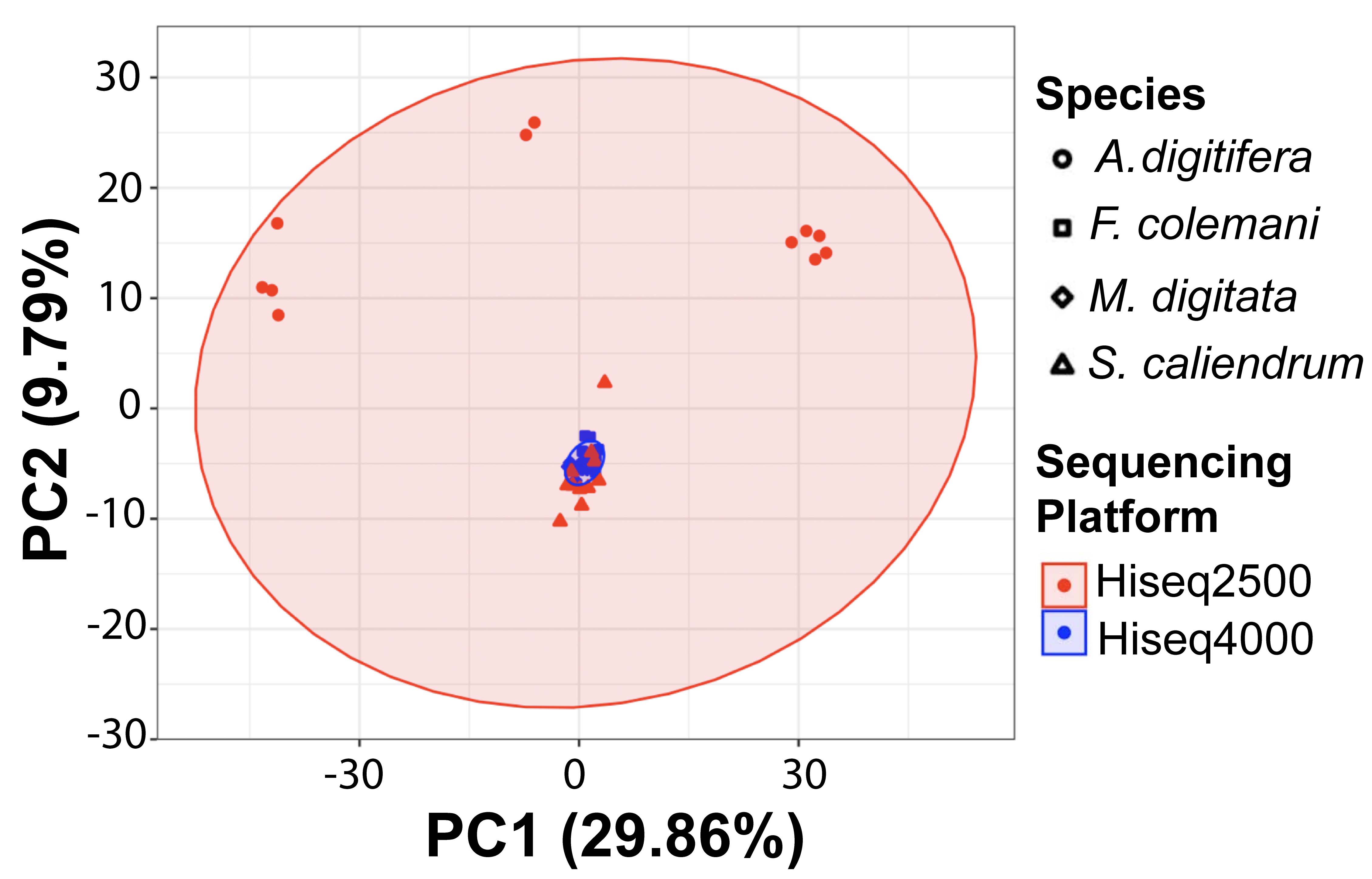

Supplement: Supplemental Information 3 — PERMANOVA revealed a significant difference based on species but no significant difference based on sequencing platform. [file peerj-12-18627-s003.jpg]

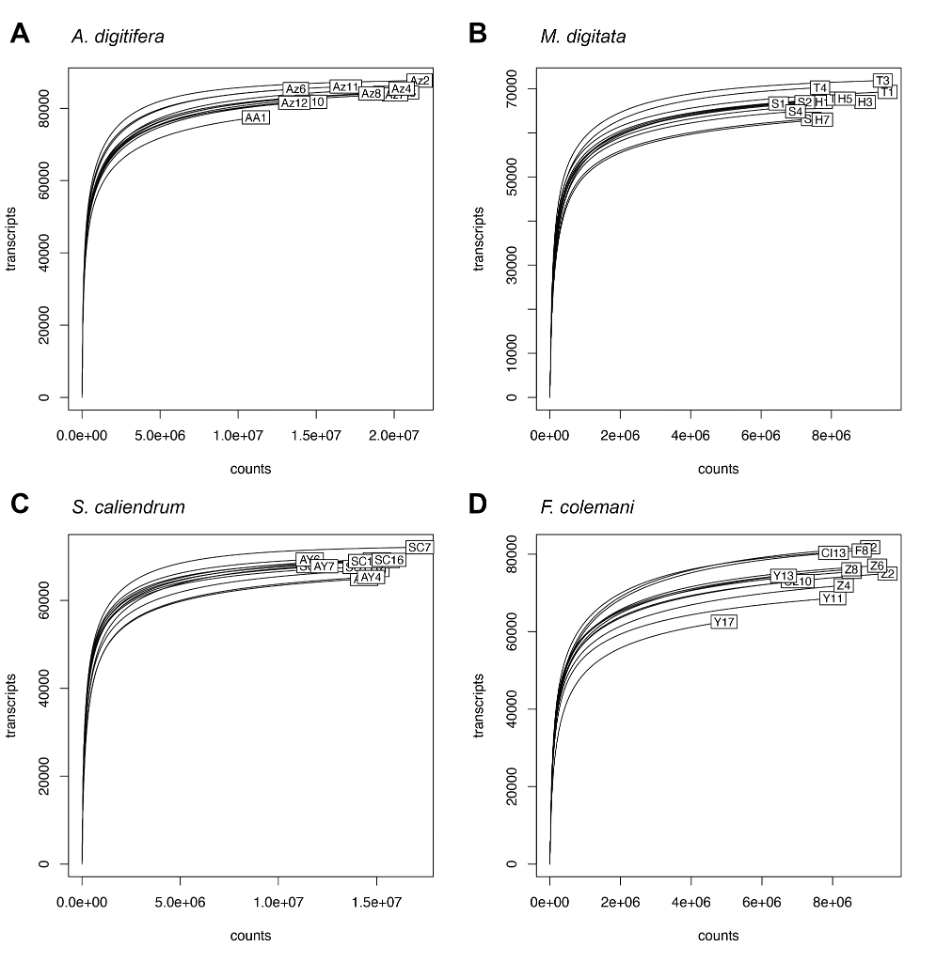

Supplement: Supplemental Information 4 — Acropora digitifera (A) and Seriatopora caliendrum (C) were sequenced on HiSeq2500, while Montipora digitata (B) and Favites colemani (D) were sequenced on HiSeq4000. Most libraries exhibit a plateau in the number of detected transcripts indicating sufficient sequencing depth. Differences in number of transcripts across holobionts (coral host and symbionts) are readily apparent, however, these are independent of the sequencing platform used and are likely species-specific signatures. [file peerj-12-18627-s004.jpg]
